# Persistent *Bartonella quintana* Infection in Macaques at Primate Research Institute, Japan

**DOI:** 10.3201/eid3210.260771

**Published:** 2026-10

**Authors:** Yoshiki Nomura, Yuka Fukudome, Akihiro Homma, Tadashi Sankai, Munehiro Okamoto, Hidenori Kabeya, Soichi Maruyama, Shingo Sato

**Affiliations:** Nihon University, Fujisawa, Japan (Y. Nomura, Y. Fukudome, A. Homma, H. Kabeya, S. Maruyama, S. Sato); Yokohama City University Graduate School of Medicine, Yokohama, Japan (T. Sankai); Kyoto University Primate Research Institute, Inuyama, Japan (M. Okamoto)

**Keywords:** Bartonella quintana, bacteria, vector-borne infections, persistent infection, primate, whole-genome sequencing, Japan

## Abstract

*Bartonella quintana*, the causative agent of trench fever, is a louseborne bacterial pathogen. During 2016−2017, we investigated *B. quintana* infection in macaques housed at 2 primate research institutes in Japan. We isolated *B. quintana* from 3 wild-caught Japanese macaques, which had been introduced into 1 institute in 2009. The macaques had received periodic ivermectin treatment every 1–2 years to control blood-feeding arthropods. Whole-genome comparisons revealed that the 3 isolates exhibit higher homology to a Japanese macaque–derived strain than to rhesus macaque–derived or human-derived strains. In addition, in all 3 isolates, the *bepA* locus was absent; we classified the *trwL* locus as structural variant C, consistent with known Japanese macaque–derived strains. Our findings suggest that *B. quintana* bacteremia in the macaques might have persisted for 7–8 years in captivity, and that the bacterium was not accidentally transmitted to them from humans by reverse zoonosis.

The genus *Bartonella* belongs to the class Alphaproteobacteria, order Rhizobiales, and family Bartonellaceae and currently comprises 49 species and 3 subspecies (*1*). *Bartonella* bacteria are transmitted among their natural hosts by blood-feeding arthropods, including fleas, lice, keds, and sand flies, which excrete the bacteria in their feces (*2*). Mammals, such as bats, cats, deer, rabbits, and rodents serve as natural reservoirs for *Bartonella* species (*3*–*6*). Vascular endothelial cells are the initial cellular targets during *Bartonella* infection, after which the bacteria are released into the host bloodstream and adhere to erythrocytes. Adherence of *Bartonella* bacteria to erythrocytes is mediated through the Trw type IV secretion system, a key determinant of host specificity among *Bartonella* species (*7*). Once established within erythrocytes, *Bartonella* bacteria are thought to persist as long-term intraerythrocytic parasites.

*B. quintana* is the etiologic agent of trench fever, a human infectious disease transmitted by the human body louse. Trench fever was first documented among Allied and German troops during World War I and sporadically reemerged during World War II (*8*). In immunocompetent persons, the disease typically manifests as relapsing fever for several days accompanied by bacteremia, severe headache, dizziness, and persistent pain in the tibias. More severe manifestations, including bacillary angiomatosis and infective endocarditis, have been reported in immunocompromised patients, particularly those with human immunodeficiency virus infection (*8*). Epidemiologic studies have demonstrated that *B. quintana* infection occurs predominantly among homeless populations in urban settings in the Americas, Europe, and Asia (*9*–*13*). Consequently, *B. quintana* is regarded as a reemerging pathogen found in modern societies.

Although those symptoms are observed in trench fever patients, *B. quintana* infection typically manifests as an asymptomatic condition in which the bacteria harbor within erythrocytes (*14*). A previous study conducted in Marseille, France, showed that 10/71 homeless persons tested positive for *B. quintana* bacteremia; 6 of those were afebrile (*10*). The remaining 4 patients exhibited prolonged bacteremia, persisting for 4–6 weeks. Asymptomatic carriers with *B. quintana* have been identified in other homeless populations in Marseille (*11*); 1 person exhibited chronic *B. quintana* bacteremia for 78 weeks, and 2 others remained bacteremic for 17 and 53 weeks. In the 1930s and 1940s, the first reports of chronic *B. quintana* bacteremia emerged among employees of laboratories producing Weigl’s type vaccines against typhus in Poland. As reported in the literature, *B. quintana* has been isolated from those employees for as long as 8 years (*15*). Subsequently, chronic bacteremia was demonstrated in experimental studies in the 1960s in which human volunteers were inoculated with the bacterium (*16*). From those findings, the duration of *B. quintana* bacteremia in the carriers is estimated to range from several weeks to ≈8 years.

Macaques inhabiting Asia have been identified as additional hosts for *B. quintana*. The first reported case of *B. quintana* infection in macaques was in a captive-bred cynomolgus macaque (*Macaca fascicularis*) imported into the United States from Vietnam (*17*). Subsequently, the bacterium was isolated from cynomolgus macaques and rhesus macaques (*M. mulatta*) born and reared at primate research centers in China (*18*,*19*) as well as from wild-caught Japanese macaques (*M. fuscata*) in Japan (*20*). In a previous study (*20*), no clinical signs were observed in the Japanese macaques positive for *B. quintana* infection, as seen in human patients with chronic *B. quintana* bacteremia; however, the duration of bacteremia in the macaques remains unknown.

A comparative genomic analysis of complete *B. quintana* genomes has revealed notable genomic differences among *B. quintana* strains from human, rhesus macaque, and Japanese macaque (*21*). Among those strains, the Japanese macaque–derived strains exhibit unique genomic features not observed in the others. Of note, strains MF1-1, MF3-1, MF10-1, MF11-1, MF19-1, and MF34-1 lack *bepA*, a gene known to confer anti-apoptotic effects in vascular endothelial cells. In addition, those strains harbor novel genomic structures within the *trwL* locus, which is implicated in erythrocyte adhesion. The human-derived strain Toulouse and the rhesus macaque–derived strain RM-11 share a conserved *trwL* structure, comprising *trwL1* to *trwL8*, in common. On the other hand, 3 distinct structural variants (A–C) have been identified among Japanese macaque strains; variant A consists of the gene order *trwL1–trwL5–trwL3–trwL7–trwL8*, variant B of *trwL1–trwLx–trwL5–trwL7–trwL8*, and variant C of *trwL1–trwLx–trwL5–trwL3–trwL7–trwL8*. The presence of *trwLx*, newly identified between *trwL1* and *trwL5* in variants B and C, highlights substantial genomic diversity within the *trwL* locus even among Japanese macaque strains (*21*).

Although the prevalence of *B. quintana* infection and the genomic properties of *Bartonella* in wild-caught Japanese macaques have been documented, its occurrence in experimental macaques controlled by human management in Japan remains unknown. In this study, we investigated *B. quintana* infection in macaque populations maintained at 2 primate research institutes in Japan. Furthermore, we performed whole-genome sequencing of *B. quintana* strains from the infected macaques to genetically characterize the *bepA* and *trwL* loci.

## Materials and Methods

### Blood Sampling

During 2016−2017, blood samples were aseptically collected from 92 wild-caught Japanese macaques and from 336 captive-bred macaques including 235 Japanese macaques and 101 rhesus macaques at the Primate Research Institute (PRI), Kyoto University (Inuyama, Japan). In 2017, additional blood samples were aseptically collected from 90 captive-bred cynomolgus macaques at the Tsukuba Primate Research Center (TPRC), National Institutes of Biomedical Innovation, Health, and Nutrition (Tsukuba, Japan) (Table 1). All blood samples were immediately stored at −70°C until further analysis.

**Table 1:** Blood sample collection of macaques in study of persistent *Bartonella quintana* infection in macaques at primate research institute, Japan*

Institute	Year	No. samples			
		Japanese macaques, wild-caught	Japanese macaques, captive-bred	Rhesus macaques, captive-bred	Cynomolgus macaques, captive-bred
PRI	2016	22	151	NA	NA
	2017	70	84	101	NA
TPRC	2017	NA	NA	NA	90
Total		92	235	101†	90‡

*NA, not applicable; PRI, Primate Research Institute, Kyoto University, Kyoto, Japan; TPRC, Tsukuba Primate Research Center, National Institutes of Biomedical Innovation, Health and Nutrition. †The macaques originated from China (n = 56) and India (n = 45). ‡The macaques originated from Indonesia (n = 30), the Philippines (n = 30), and Malaysia (n = 30).

The wild-caught Japanese macaques originated from several geographic locations and were transferred to PRI as follows: 8 macaques from Aichi Prefecture, in August 2003; 15 from Shizuoka Prefecture, in June 2008; 22 from Wakayama Prefecture in February 2009; and 47 from Osaka Prefecture during March 2008–June 2009. Upon arrival at the facility, all wild-caught macaques underwent deworming treatments with ivermectin. The ivermectin solution Ivermec Inj (Fujita Pharmaceutical Co., http://www3.fujita-pharm.co.jp) was administered subcutaneously at a dose of 0.1 mL/kg. In addition, both wild-caught and captive-bred macaques received periodic ivermectin treatments during routine physical examinations conducted at intervals of 1–2 years. At PRI, macaques were kept outdoors in social groups according to their geographic origin. In contrast, macaques at the TPRC were housed indoors in individual cages and received medicated baths with the organophosphorus ectoparasiticide Neguvon (Bayer, https://www.bayer.com) at 2-year intervals.

All animal procedures were conducted in accordance with applicable animal welfare regulations. Blood sampling at the PRI was performed under approved animal experimentation protocols (approval nos. 2016-158 and 2017-089). Blood samples from the TPRC were provided through the Nonhuman Primate Reagent and Resource Program under an institutional agreement for the distribution of research materials.

### Detection of *Bartonella* DNA from Macaque Blood Samples

To screen for *B. quintana* infection in the macaque populations, we performed nested PCR targeting the RNA polymerase β-subunit gene (*rpoB*) of the genus *Bartonella*. We designed the first PCR primers in this study, 1913f and 2357r, based on the *rpoB* sequences from *B. quintana* strains MF1-1 and RM-11. We designed the second PCR primers, 600f-m and 800r-m, with some modification based on original primers (*22*) (Table 2). The positive control was genomic DNA extracted from *B. quintana* strain MF1-1, whereas the negative control was genomic DNA extracted from a blood of a cynomolgus macaque classified as SPF grade. Conditions for the nested PCR were as follows: 2 minutes at 94°C followed by 35 cycles of 30 seconds at 94°C, 30 seconds at 54°C, and 1 minutes at 72°C, and a final extension of 2 minutes at 72°C. We purified the second PCR amplicon using a Wizard SV Gel and PCR Clean-Up System (Promega Corporation, https://www.promega.com), followed by direct DNA sequencing with the Genetic Analyzer models 3730xl (Thermo Fisher Scientific, https://www.thermofisher.com). We compared the obtained *rpoB* sequences with those in the International Nucleotide Sequence Database using the BLAST (https://blast.ncbi.nlm.nih.gov/Blast.cgi).

**Table 2:** Primer information for nested PCR targeting the *rpoB* gene used for study of persistent *Bartonella quintana* infection in macaques at primate research institute, Japan*

Use application	Primer	Nucleotide sequences, 5′ → 3′	Amplicon size, bp	Reference
1st PCR	1913f	ACGCTGAGGGACGTTTTACAG	445	This study
	2357r	ACATGCACGAGAGGACGCTG		
2nd PCR	600f-m	GAAAATGATGATGCRAATCG	211	22
	800r-m	GATCTAAATCTTCTGTRGCACG		

### Isolation and Identification of *Bartonella* Bacteria from Macaque Bloods

We isolated *Bartonella* bacteria as described previously (*20*). In brief, we spread a 100 µL of freeze-thawed blood samples onto 5% rabbit blood chocolate agar plates and incubated them at 35°C under 5% CO_2_ for up to 4 weeks. We tentatively identified colonies as *Bartonella* species on the basis of characteristic morphology (small, round, gray to cream-yellow colonies) and the requirement for a long culture period (>1 week). We calculated colony-forming units (CFUs) per milliliter of blood. We recovered 5 colonies from each culture-positive sample and subcultured them on fresh chocolate agar plates under the same conditions as the primary culture.

We extracted genomic DNA from each isolate using InstaGene Matrix (Bio-Rad Laboratories, https://www.bio-rad.com). We performed species identification by *Bartonella*-specific PCR targeting the *rpoB* (*23*), citrate synthase (*gltA*) (*24*), and 16S–23S rDNA intergenic transcribed spacer (ITS) regions (*25*). We used genomic DNA from *B.* *alsatica* strain IBS382^T^ as the positive control and nuclease-free distilled water as the negative control. We purified and sequenced PCR amplicons and compared them with the corresponding sequences derived from *B. quintana* strain MF1-1 (RefSeq [https://www.ncbi.nlm.nih.gov/refseq] accession no. NZ_AP019773).

### Whole-Genome Sequencing Analysis

We extracted genomic DNAs from representative strains using DNeasy Blood and Tissue Kit (QIAGEN, https://www.qiagen.com). We prepared sequencing DNA libraries from the extracted genomic DNAs by using Illumina DNA Prep and IDT for Illumina DNA-RNA UD Indexes Set A, Tagmentation (Illumina, https://www.illumina.com). We sequenced the DNA libraries on a MiSeq platform using the MiSeq Reagent Nanokit version 2 (500 cycle) (Illumina). We quality-checked raw reads and assembled them using a de novo assembler program, the strategic K-mer extension for scrupulous assemblies (*26*), to generate contigs. We assessed de novo assembly qualities using Benchmarking Universal Single-Copy Orthologs (BUSCO) version 5.4.3 (*27*).

We assessed nucleotide sequence identities at whole-genome levels by pairwise average nucleotide identity (ANI) (*28*) and digital DNA-DNA hybridization (dDDH) (*29*). We compared the assemblies of representative strains with strains MF1-1, RM-11, and Toulouse.

We confirmed the presence or absence of the *bepA* locus comprising *bepA1* and *bepA2* by *bepA*-specific PCR and subsequently validated by whole-genome sequencing data. We identified structural variants within the *trwL* locus by comparison with previously reported data (*21*).

### Data Availability

We deposited raw reads of all the sequenced *B. quintana* strains in this study in the Sequence Read Archive of the DNA Data Bank of Japan. All are available under BioProject accession no. PRJDB40655.

## Results

### Prevalence of *B. quintana* in Macaque Populations at PRI and TPRC

We confirmed *Bartonella* infection by the *rpoB*-targeting PCR in 3 (3.3%) (sample IDs TB1, MN51, and MN57) of the 92 wild-caught Japanese macaques housed at PRI. All PCR amplicons showed an identical sequence to the *rpoB* of strain MF1-1. In contrast, we detected no *Bartonella* DNA in the captive-bred macaques at either the PRI or the TPRC.

We isolated *Bartonella* bacteria from the 3 PCR-positive Japanese macaques but not from any of the captive-bred macaques we examined. Bacteremia levels were 2.9 × 10^3^ CFU/mL for TB1, 4.0 × 10^2^ CFU/mL for MN51, and 3.0 × 10^3^ CFU/mL for MN57. We recovered a total of 15 *Bartonella* isolates, 5 isolates per *Bartonella*-positive macaque. All the isolates showed 100% sequence identity for the *gltA* and *rpoB* and 99.9%–100% sequence identity for the ITS, compared with those of strain MF1-1.

According to management records at PRI, macaque TB1 was captured in Wakayama Prefecture and transferred to the PRI in February 2009. Similarly, macaques MN51 and MN57 were captured in Osaka Prefecture and transferred to PRI in June 2009. Blood samples for this study were collected in November 2016 from TB1 and in November 2017 from MN51 and MN57.

### De Novo Assemblies Using Whole-Genome Sequences

We randomly selected 1 representative strain from each culture-positive macaque, yielding strains TB1-1, MN51-1, and MN57-1 for whole-genome sequencing analysis. The number of sequencing reads generated by the MiSeq platform ranged from 303,794 for strain MN51-1 to 1,065,204 for strain MN57-1 (Table 3). De novo assembly produced 43 contigs for strain TB1-1, 52 contigs for strain MN51-1, and 47 contigs for strain MN57-1; average genome coverage exceeded 46×. We estimated genome sizes of the 3 strains on the basis of the assembled contigs as ≈1.6 Mbp with guanine-cytosine contents of 38.8% for all strains. BUSCO scores of the 3 assembled contigs were 97.8–97.9.

**Table 3:** Whole-genome sequencing data of 3 *Bartonella quintana* strains from wild-caught Japanese macaques kept in primate research institute in study of persistent *B. quintana* infection in macaques at primate research institute, Japan*

Strain	No. short reads	Total bases calculated from all reads	No. contigs	Coverage†	Estimated genome size, mbp	GC, %	BUSCO score, %
TB1-1	700,410	166,315,725	43	106-fold	1.6	38.8	97.8
MN51-1	303,794	73,058,573	52	46-fold	1.6	38.8	97.9
MN57-1	1,065,204	258,705,960	47	165-fold	1.6	38.8	97.9

*BUSCO, benchmarking universal single-copy orthologs; GC, guanine–cytosine contents. †Indicates increase over average coverage.

We compared whole-genome sequences by ANI and dDDH. We compared the 3 representative strains with strain MF1-1 from a Japanese macaque, strain RM-11 from a rhesus macaque, and strain Toulouse from a human (Table 4). The ANI values obtained from the comparison of the representative 3 strains with strain MF1-1 were 99.8%, higher than those against strain RM-11 (99.4%) and strain Toulouse (98.3%). Similarly, the dDDH values obtained from comparing the representative strains with strain MF1-1 were 98.9%–99.0%, which exceeded those against strain RM-11 (95.3%–95.4%) and strain Toulouse (85.2%–85.3%).

**Table 4:** Whole-genome comparisons based on ANI and dDDH values in study of persistent *Bartonella quintana* infection in macaques at primate research institute, Japan*

Strain	Host	% ANI value/dDDH value		
		TB1-1	MN51-1	MN57-1
MF1-1	Japanese macaque	99.8/99.0	99.8/98.9	99.8/98.9
RM-11	Rhesus macaque	99.4/95.4	99.4/95.3	99.4/95.3
Toulouse	Human	98.3/85.2	98.3/85.3	98.3/85.3

*ANI, pairwise average nucleotide identity; dDDH, digital DNA-DNA hybridization.

### Genomic Characterization of the *bepA* and *trwL* Loci

The *bepA* locus (1,612bp) was absent from all 3 representative strains (Figure 1), which we confirmed by the whole-genome sequencing data. Gene synteny analysis revealed that the *trwL* locus in the representative strains corresponded to structural variant C, characterized by the gene order of *trwL1*, *trwLx*, *trwL5*, *trwL3*, *trwL7*, and *trwL8* (Figure 2).

**Figure 1:**
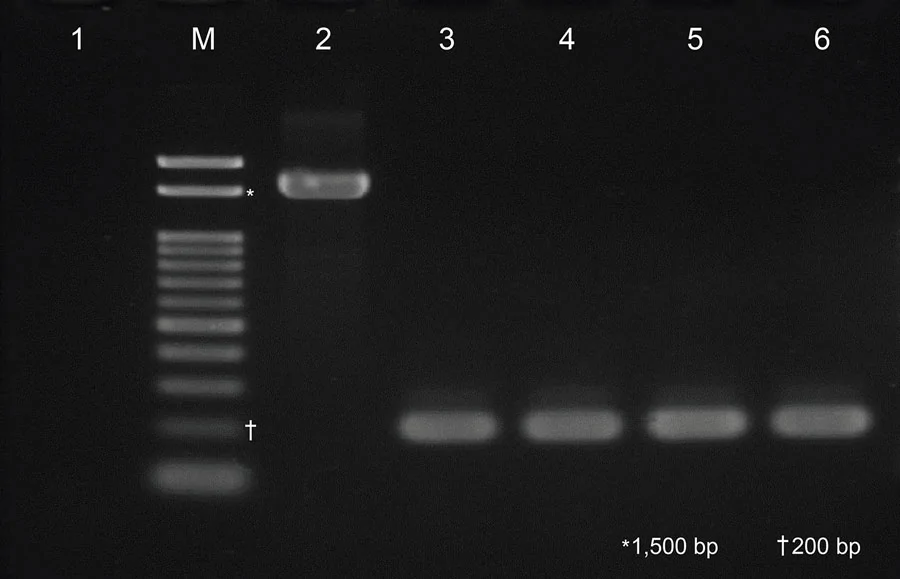
Electrophoretic analysis of *bepA*-specific PCR amplicons in study of persistent *Bartonella quintana* infection in macaques at primate research institute, Japan. Lane M, 0.1–2 kb DNA size marker; lane 1, nuclease-free water (negative control); lane 2, *Bartonella quintana* strain Fuller^T^ (positive control); lane 3, strain MF1-1; lane 4, strain TB1-1; lane 5, strain MN51-1; lane 6, strain MN57-1. The expected amplicon size for the *bepA* locus is 1,612 bp, as observed in the positive control (lane 2). As with strain MF1-1 (lane 3), amplicon size of strains TB1-1, MN51-1, and MN57-1 (lanes 4–6) was ≈200 bp, indicating the absence of the *bepA* locus in the chromosome.

**Figure 2:**
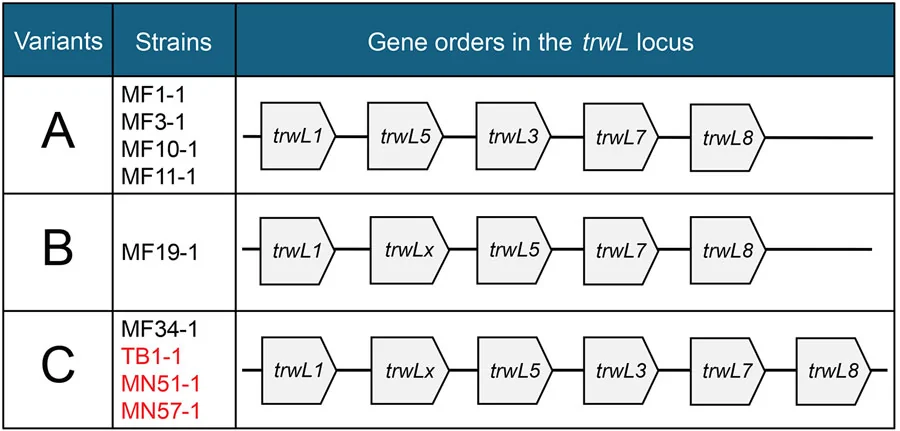
Structural variants of the *trwL* locus among *Bartonella quintana* strains from Japanese macaques in study at primate research institute, Japan. We reviewed 3 structural variants (A–C) of the *trwL* locus as previously described (*21*). Red text indicates representative strains analyzed in this study, which were classified as variant C.

## Discussion

In this study, we isolated *B. quintana* in a research colony of 3 Japanese macaques housed at the PRI, all of which originated from wild-caught populations. The bacteremia levels (400–3,000 CFU/mL) were comparable to those previously reported in Japanese macaques (*20*). In contrast, no *Bartonella* infection was detected in any of the captive-bred Japanese, cynomolgus, or rhesus macaques housed at either of the 2 primate facilities. Although both wild-caught and captive-bred macaques at the 2 facilities received periodic antiparasitic treatments, the *B. quintana*–positive Japanese macaques might have been infested with some kind of blood-feeding arthropods, such as monkey lice (*Pedicinus* sp.) (*30*), before they were captured in their habitats. We found monkey lice on wild-caught Japanese macaques in Kanagawa Prefecture, Japan, during 2018–2022 and successfully detected *B. quintana* DNA from the lice (data not shown). Because the *B. quintana*–positive Japanese macaques were introduced into the PRI in 2009 and tested positive for *B. quintana* infection in 2016–2017, we presumed that they maintained persistent *B. quintana* infection for up to 8 years. Similarly, in 1949, *B. quintana* bacteremia was documented as persisting for up to 8 years in trench fever patients (*15*). On the basis of all those findings, we concluded that *B. quintana* can establish long-term persistent infections in both humans and nonhuman primates. Such long-term, asymptomatic *B. quintana* bacteremia demonstrates that infected individuals can act as chronic carriers of the pathogen without manifesting distinct clinical symptoms. However, we noted that chronic bacteremic states in humans might eventually progress to severe complications, such as endocarditis (*31*).

We performed subsequent whole-genome sequencing to genetically characterize the 3 representative strains TB1-1, MN51-1, and MN57-1 from the *B. quintana*–positive Japanese macaques. We evaluated genome assembly quality using BUSCO analysis, which yielded completeness scores >95% for all strains (*27*), supporting the reliability of the de novo assemblies. In addition, the estimated genome sizes and guanine-cytosine contents were highly consistent with those reported for other known *B. quintana* strains (*21*,*32*), indicating that the assembled genomes were suitable for downstream comparative analyses. Comparisons at the whole-genome level using ANI and dDDH revealed that the 3 strains exhibit higher homology to strain MF1-1 from a Japanese macaque (*21*) than to strain RM-11 from a rhesus macaque and strain Toulouse from a human. Of note, in all 3 strains, the *bepA* locus was absent, and the *trwL* locus was classified as structural variant C; we had demonstrated previously that both loci seem to be unique to Japanese macaque–derived strains (*21*). Those findings suggest that the *B. quintana*–positive Japanese macaques did not accidentally acquire the bacterium from humans within this facility, ruling out the likelihood that reverse zoonosis caused the infections. Future surveillance of *B. quintana* infection in other nonhuman primates, such as apes and New World monkeys, will increase understanding of the host range of the bacterium in nature.

Studying infection state in the same animals over time would be helpful to further confirm long-term persistent infection. However, the PRI ethics guidelines restrict blood sampling from the same macaques to once every 2 years. Furthermore, the macaque TB1 died in June 2018 from an accident within the PRI enclosure, which prevented the collection of consecutive blood samples. Although we attempted to collect additional blood samples from macaques MN51 and MN57 since November 2019, experiments at PRI were suspended for several years during the COVID-19 pandemic, making subsequent medical research applications exceedingly challenging. To address those issues, we would need to conduct infection experiments for *Macaca* spp. monkeys with Japanese macaque–derived strains in another primate research facility.

In conclusion, we identified *B. quintana* bacteremia in 3 wild-caught Japanese macaques housed at PRI and noted evidence suggesting persistent infection for up to 8 years after capture. Given that the *trw* operon, particularly the *trwL* and *trwJ* loci, plays a crucial role in erythrocyte adhesion (*7*), further investigation is warranted to determine whether structural variations in *trwL* influence the ability of macaque-derived *B. quintana* strains to infect human erythrocytes. Such studies would be particularly relevant from One Health perspective. Our findings highlight the capacity of *B. quintana* bacteria to establish long-term infection in Japanese macaques and support previous documentation of prolonged bacteremia in humans.
